# Is She a Nurse or Not?

**Published:** 1919-11-01

**Authors:** 


### THE STATUS OF THE MIDWIFE.

### Is She a Nurse or Not?

Is a Certified Midwife a Nurse, or is she not? This question has been raised in Ireland, and before it is satisfactorily answered interesting developments may be expected to ensue.

During the war it will be remembered that Sir Arthur Stanley came to Ireland to inaugurate there the Nation's Tribute to Nurses. He gathered around him an enthusiastic band of sympathisers, and addressed large and influential public meetings in Dublin and Belfast. Everybody that was anybody gave him their whole-hearted support, and a sympathetic public responded. The result was a collection of £10,200. It is a historical fact which cannot be denied that but for Sir Arthur Stanley there would be no Irish Tribute for the nurses' benefit. When it was an assured success the Pussyfoot sisterhood came along, and, having up to that point derided the movement as an "insult" to the "independent" nurse, proceeded by subtlety and stealth to capture and administer the Fund. Sir Arthur was denounced as "English," a person trying to bleed the unfortunate Irishry, and to carry off the spoil for distribution in Great Britain. "This is Irish money, and must be spent in Ireland and administered in Ireland." So ran the war-cry of a clique of women who in their lives had never raised a finger or an eyelid for the betterment of the profession. The sorry part of the story is that they succeeded. When the Fund was complete Sir Arthur Stanley was put outside the door, and, needless to observe, the chivalrous heroines who accomplished this triumph have not since added, or attempted to add, a farthing to the Fund. Their function is to distribute.

### When the Question Arose.

It was at a meeting of the Irish Tribute Executive that the question arose as to whether the Midwife is or is not a Nurse, under the meaning of the Act, as the lawyers would put it. If a midwife finds herself in distress, is she or is she not entitled to apply to this Fund for relief? The matter was duly discussed, and, as there appeared to be a difference of opinion, Miss Huxley proposed, and Miss Carson Rae seconded, a resolution that midwives should not be permitted to participate. This resolution was carried almost unanimously. The Nurses' Union—a Trade Union—dispute the justice of the finding, and the question is now being discussed in the daily press of Ireland. Considering that there are two thousand trained midwives registered in Ireland, and that their services are of vital importance in the matter of public health administration, it is not probable that the last word has yet been said on the subject, nor is it apparent that public opinion will endorse the action of the Irish Nurses' Tribute Executive.

It is, I suggest, obvious that the first duty of any Committee administering funds subscribed by the public is to interpret correctly the wishes of the public. There is no question of relative professional status. The public had no such distinctions submitted for their consideration. The relief of aged, necessitous or infirm "nurses," trained and certified, was the case to which they were asked to respond.

The principal argument in favour of the Resolution would appear to be that the income derived from the investment of the capital is too small to share with Midwives. £500 a year is the gross income, and secretarial and office expenses consume one-third of this, or thereabouts. Had Sir Arthur Stanley remained in possession it is obvious that this waste pipe would not have been flowing. The machine for distributing benefits in Great Britain would have discharged the Irish function at a nominal cost or altogether free. Ireland's claims could easily have been earmarked and insured, and the recommendations of a local Committee adopted as to distribution. Any lawyer, accountant, or business man would see with half an eye that centralisation and economy run hand in hand. No man in the kingdom is better aware of this fact than Sir Arthur Stanley. The outstanding phenomenon of Red Cross administration under his chairmanship was that every shilling subscribed was spent on the wounded. The cost of administration was covered by bank interest. Ireland subscribed £10,000 for Nurses, and some £160 of the £500 interest on the Fund is used up in office expenses. Necessitous nurses are losing precisely this sum, and the remedy provided by the Committee of Management is the expropriation of the Certified and Trained Midwife.

### The Evolution of the Nurse.

I confess my surprise that Miss Huxley and Miss Carson Rae should be the apostles of this reactionary movement. They are the high priestesses of the Irish Nurses' Association, which for many years has been receiving subscriptions from Midwives and Maternity Nurses, presumably for their benefit and advancement. The smallness or greatness of the income derived from the Tribute Fund cuts no figure in what is purely a matter of principle. I venture to contend that the Trained Midwife is a very important member of the Nursing profession, and I venture further to point out that she was a substantial factor in the collection of the Irish Tribute. I remember attending a Red Cross entertainment in the Theatre Royal, Dublin, an enterprise for the collection of funds. One of the most attractive features was a series of tableaux illustrating the evolution of the Nurse. It was splendidly staged, and, literally, "brought down the house." The most prominent feature of this display consisted of tableaux showing "Sairey Gamp" with her black bottle, and showing in striking contrast the modern maternity nurse, queen of the sick-room. If my recollection serves me aright, one, if not both, of the ladies who now propose the exclusion of the Midwife from participation in the Tribute Fund were active workers in preparing this money-winning appeal. An explanation of their attitude, past and present, would be interesting and illuminating.

The whole subject demands the immediate and urgent attention of the leading spirits of the nursing world. The Midwife is the only State Registered Nurse in the kingdom. With additional qualifications she promises to be an essential factor in the reconstruction schemes of the Ministry of Health. In Ireland a small coterie, very select and very autocratic, have offered her what she regards as a serious affront, and the matter is being taken in hand by a particularly active Nurses' Trade Union.

These are the facts, and it would be folly to adopt an ostrich policy in the matter.

